# On the efficacy of per-relation basis performance evaluation for PPI extraction and a high-precision rule-based approach

**DOI:** 10.1186/1472-6947-13-S1-S7

**Published:** 2013-04-05

**Authors:** Junkyu Lee, Seongsoon Kim, Sunwon Lee, Kyubum Lee, Jaewoo Kang

**Affiliations:** 1 Department of Computer Science, Korea University, Seoul, Korea

## Abstract

**Background:**

Most previous Protein Protein Interaction (PPI) studies evaluated their algorithms' performance based on "per-instance" precision and recall, in which the instances of an interaction relation were evaluated independently. However, we argue that this standard evaluation method should be revisited. In a large corpus, the same relation can be described in various different forms and, in practice, correctly identifying not all but a small subset of them would often suffice to detect the given interaction.

**Methods:**

In this regard, we propose a more pragmatic "per-relation" basis performance evaluation method instead of the conventional per-instance basis method. In the per-relation basis method, only a subset of a relation's instances needs to be correctly identified to make the relation positive. In this work, we also introduce a new high-precision rule-based PPI extraction algorithm. While virtually all current PPI extraction studies focus on improving F-score, aiming to balance the performance on both precision and recall, in many realistic scenarios involving large corpora, one can benefit more from a high-precision algorithm than a high-recall counterpart.

**Results:**

We show that our algorithm not only achieves better per-relation performance than previous solutions but also serves as a good complement to the existing PPI extraction tools. Our algorithm improves the performance of the existing tools through simple pipelining.

**Conclusion:**

The significance of this research can be found in that this research brought new perspective to the performance evaluation of PPI extraction studies, which we believe is more important in practice than existing evaluation criteria. Given the new evaluation perspective, we also showed the importance of a high-precision extraction tool and validated the efficacy of our rule-based system as the high-precision tool candidate.

## Background

The volume of new biomedical literatures available for processing rapidly increases. Currently more than 2000 new articles are added daily to the Medline database. As it became obvious that purely manual curation cannot cope with the fast growing data, attention has been increasingly directed to the automatic information extraction techniques from the BioNLP and Bio-text mining communities. The protein-protein interaction (PPI) extraction problem is the most extensively studied information extraction problem in the biomedical domain.

PPI extraction research is largely categorized into two groups based on the types of classification models they use. One group of approaches use rules and patterns to describe the matching protein pairs [1-4]. The others rely on machine learning methods to predict the interaction pairs [5-8]. New methods are consistently introduced, improving extraction performance. However, we argue that there is an inherent and grossly ignored problem in the performance evaluation methods employed in the research.

First, virtually all PPI extraction research focuses on improving F-score, which gives equal emphasis on both precision and recall. Depending on the application scenario, we may have to value one more than the other. For example, suppose we are to construct a knowledge base for biomedical events by combining manually curated sources together with automatically extracted information through literature mining. In such a case, one might prefer a high-precision PPI extraction tool to its high-recall counterpart because potential false positive relations introduced by the high-recall tool will significantly degrade the accuracy and reliability of inferred knowledge from the combined database. In spite of this, almost all existing PPI research focus on improving F-score, giving equal weights on both precision and recall.

Second, current PPI research evaluates their performance on a "per-instance" basis. For example, in AIMed corpus [9], the IL-6 and gp130 pair appears total of 29 times, 8 instances annotated as positive (i.e., "interaction") and the remaining 21 annotated as negative. According to the conventional per-instance basis evaluation, 100% accuracy is achieved only when a PPI tool correctly labels all the 8 positive instances as positive and the remainders as all negative. However, in principle, the 8 positive instances describe the same relation, IL6/gp130 interaction. They merely describe it in different linguistic styles, some straightforward and some others complex and/or indirect. Hence, correctly identifying any one of them would be helpful.

The "non-interaction" cases aggravate the situation. In AIMed, the PTF/TBP pair is annotated 26 times, all as negative. Just a single mistake will make the PTF/TBP pair an "interaction," i.e., a false positive, or at least put it in limbo where human intervention is required in order to draw a definitive conclusion. The false positive problem aggravates as the size of corpus increases. Note that the AIMed corpus consists of 225 abstracts containing only 1955 sentences. The real-world corpus (e.g., PubMed) which we have to deal with in practice, is substantially larger than the benchmark corpora. This strongly suggests that, although it has been grossly ignored so far by the researchers developing PPI tools, an ultra-high precision PPI extraction tool can be extremely useful to domain scientists who actually use it in many application scenarios.

Given these observations, we introduce a new performance evaluation method based on "per-relation" basis instead of the conventional per-instance basis, which is more pragmatic in practice. We also introduce a new pattern-based PPI extraction method that achieves extremely high precision while retaining good recall in the new per-relation basis evaluation. We have reported the preliminary performance results of our rule-based algorithm in [10]. In this work, we generalize our algorithm into a two-tier framework. With this framework, our rule-based algorithm can be combined with other PPI solutions through simple pipelining. For example, we can use an existing high-performance extraction algorithm in the first phase and then pipeline the results to our high-precision rule-based algorithm for the second screening.

We expect that our method is more practical in real-world applications than conventional methods that are designed to balance the per-instance basis precision and recall. We validate our method using the AIMed corpus, a widely used benchmark corpus for PPI extraction tasks.

### Related work

PPI extraction research is largely categorized into two approaches: pattern-based and machine learning-based approaches. We briefly survey the two methods below.

#### Pattern-based PPI extraction

Pattern-based methods define lexical and/or syntactic patterns to find matching text regions that are likely to contain PPIs. Many of early PPI systems fall into this category [11-13]. Blaschke et al used a pre-defined set of 14 verbs indicating interactions and composed a series of rules based on the verb and protein arrangement [11]. Ono et al defined simple POS rules for matching interactions [13]. They also employed regular expression patterns to filter negative sentences in order to reduce false positives. Huang et al proposed a method for automatically generating patterns for PPI extraction [12]. They used a dynamic programming algorithm to compute discriminative patterns by aligning sentences and key verbs that describe interactions. Finally, a matching algorithm is proposed to evaluate the patterns.

More recently, approaches utilizing computational linguistic technologies have been introduced [1,2,4]. These methods use parsing techniques to make the patterns more precise and systematic. The methods are further divided into two categories: shallow parsing-based [1,3] and deep parsing-based [2,4]. Parsing is a very computationally demanding NLP task. The shallow parsing-based methods tradeoff accuracy for computational efficiency. In [1], Ahmed et al split complex sentences into simple clausal structures made up of syntactic roles, tagged biological entities using ontologies, and finally extracted interactions by analyzing the matching contents of syntactic roles and their linguistic combinations. Meanwhile, Fundel et al proposed an extraction system, RelEx, employing more sophisticated rules utilizing "full" parse tree structures [2]. Rinaldi et al also employed a probabilistic dependency parser to extract patterns describing biological interactions [4].

These pattern-based methods achieve good performance with respect to F-score. However, because of the coarsely defined rules, they produce large numbers of false positives, making them inapplicable to use cases where "per-relation" precision is important.

#### Machine learning-based PPI extraction

Recently, many machine learning-based approaches have employed linguistic engineering techniques including shallow and full parsing. Among them, kernel-based methods have been investigated most extensively [5-8]. They typically parse a sentence containing a protein pair and extract some lexical and syntactic features from the parsing result. Depending on the approaches, the extracted features either are vectorized to be used with conventional kernel functions such as RBF and polynomial kernels [6,14,15], or are used as is as input to a custom-designed kernel [5,8,16].

Many of the recently introduced custom kernels are convolution kernels [17]. Convolution kernels take as input two discrete structures such as strings, trees, and graphs, and compute their similarity by recursively aggregating the similarity of their "parts." In our context, some relevant parts of the parse tree or lexical subsequences can be used as the input to the convolution kernels. Kernel methods in this category include subsequence kernels [18,19], tree kernels [20,21], and shortest path kernels [9]. Please see [22] for a more comprehensive survey and benchmark study for the kernel-based methods.

**Data**: A protein pair to be tested, sentence/clause, dependency tree

**Result**: Positive, if interaction exists; Negative, otherwise

**foreach ***rule R_i _from R*_1 _*to R*_8 _**do**

  test if the input pair matches *R_i_*;

  **if ***matched ***then**

    return Positive;

  **end**

end

Return Negative;

**Algorithm 1: **Rule-based PPI classifier

Although the machine learning-based approaches generally achieve better performance than the pattern-based approaches, they still face the same problem. Because of the inherent probabilistic nature of the machine learning methods, it is very difficult to design an ultra-high precision machine-learning based classifier. For this reason, the machine learning-based approaches also are not appropriate for our problem context. In this work, we address these problems by introducing a rich set of high precision lexical/syntactic rules.

## Methods

Our rule-based system works in two steps: text preprocessing/parsing and PPI rule evaluation for extraction. We explain the details for the two steps below.

### Text preprocessing and parsing

Recent study shows that the accuracy of a parser has a non-negligible impact on the accuracy of PPI extraction tasks [23]. As no parser can be perfect, we preprocess the text in order to reduce the potential risk of parser errors. In this work, we follow the preprocessing procedure used in [6]. First, we replace the protein names with *PROTEIN*0, 1, 2, etc., in order to replace a complex multi-word protein name with a single term. This practice is commonplace in many PPI extraction studies because comprehensive protein name dictionaries are available and the focus of the study is to find the relations, and not the entity recognition [8,22]. Second, we remove parentheses and the enclosed words if no protein exists within the parenthetical remark. Third, sentences consisting of multiple clauses are split into separate clauses, and finally, only the sentences/clauses containing at least two proteins are analyzed with the Stanford parser to produce the dependency tree [24].

### Rule-based PPI extraction

We model the PPI extraction task as a binary classification problem. For each protein pair within a sentence, all rules are applied in sequence until a matching rule is found. The outline of our rule-based PPI classification is given in Algorithm 1. Given a candidate protein pair, a sentence containing the pair, and its dependency tree, the algorithm returns positive if a matching rule is found, and negative otherwise.

We use a total of eight rules in our framework. The first three are the refined versions of the rules introduced originally in the RelEx system [2]. The rest are the newly introduced rules in this work. We explain the rules using examples below.

***Rule 1:*** *P_i _- REL - P_j_*

**Figure 1:**
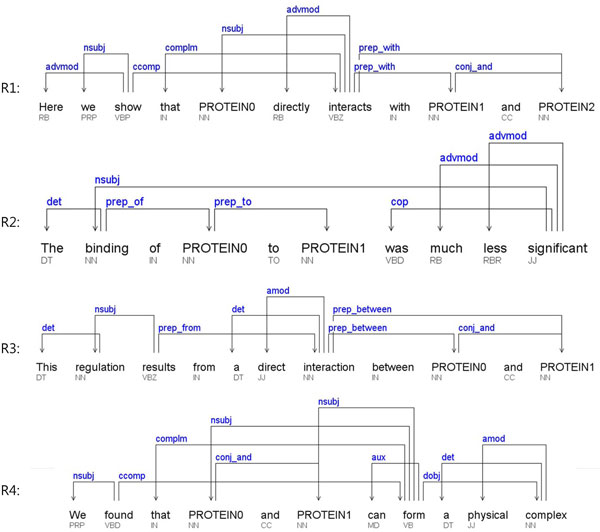
**Dependency relations for the example sentences**.

An example sentence and its dependency relations are illustrated in Figure 1-R1. In this example instance, two PPIs exist: *P*0 *- P*1 and *P*0 *- P*2. This rule is intended to capture a pattern where the first protein is the nominal subject (*nsubj*), verb corresponds to a relation word, and the second protein is either a direct object (*dobj*) or a prepositional modifier (prep* ) of the verb. In the example, *P*0 is the subject of "interacts," a relation word, and P1 is the prepositional modifier of "interacts," and hence *P*0 *- P*1 matches the rule. *P*0 *- P*2 is also extracted by the same rule.

Note that we also handle negation by checking if a negation (*neg*) dependency exists on either the relation word or the subject. For example, in a sentence, "*P*0 does not bind to *P*1," no PPI is extracted because there exists a *neg *dependency from "bind" to "not." As for the relation words, we compiled 67 keywords that clearly describe various types of interactions between a pair of proteins. The relation keywords are shown in Table 1.

**Table 1:** Relation keywords

Activation	activate, increase, induce, initiate, stimulate, promote, catalyze, up-regulate, coactivate, potentiate, precipitate, reactivate
Deactivation	block, decrease, down-regulate, inactivate, inhibit, reduce, repress, suppress, interfere, antagonize, degrade, obstruct

Creating bond	associate, link, dimerize, heterodimerize, crystalize, methylate, phosphorylate, assemble, polymerize, bound, bond, oligomerize, glycosylate, bind, complex, form, conjugate, acetylate, couple

Breaking bond	cleave, demethylate, dephosphorylate, sever, unbind, depolymerize, dissociate, deacetylate, deglycosilate, disassemble

Signaling	mediate, modulate, participate, regulate, control, interact, react, contact, response, encode, recognize, stabilize, destabilize, target

If the parsing result is always correct, the rule should extract correct PPIs all the time. However, it is far from reality. Long-distance relations in complex sentences are typically prone to parsing errors. The long-distance relations are also susceptible for mistakes by human annotators due to the high complexity of sentences. As we are specifically aiming for an extreme high-precision PPI extraction method, we ignore such long-distance relations. We achieve this by considering only the pairs within a *seven*-word window on the original sentence. We also put a constraint on the maximum number of dependency-bearing words allowable in between the two proteins. We empirically determined the max to be *three*. In the example, *P*0 *- P*2 is a valid candidate because the two proteins fall within a seven-word window and there are only three dependency-bearing words in between them (i.e., "with" and "and" do not count).

***Rule 2:*** *REL - of - P_i _- PREP - P_j_*

This rule is intended to match a phrase like "binding of *P*0 to *P*1" as shown in the example in Figure 1-R2. We first find a relation word "binding" and follow the *prep_of *dependency to retrieve the first protein *P*0. We then follow a prep* dependency to capture the second protein *P*1. Other examples that match this rule include "activation of *P*0 by *P*1" and "interaction of *P*0 with *P*1." The negation handling in this case is done in two different places. First, as in the example, "no interaction of *P*0 with *P*1 was found," the negation (*neg*) dependency must be checked on the relation word ("interaction"). Second, the negation also can occur at verb level as in the following example: "activation of *P*0 by *P*1 was not identified."

***Rule 3: ****REL - *{***between, of***} *- P_i _- and - P_j_*

We first find a relation keyword and follow the *prep_between *or *prep_of *dependencies from the keyword to retrieve two proteins. In the example in Figure 1-R3, there are two relation keywords ("regulation" and "interaction") in the sentence but only the second ("interaction") matches the rule. The negation handling is performed in a similar way as Rule 2.

***Rule 4:*** *P_i _- and - P_j _- REL*

This rule covers the cases where a protein conjunction (*P*0 and *P*1) is involved in *nsubj *dependency and the conjunction is linked to a relation keyword. However, not all relation keywords are acceptable in this rule. The rule rejects the PPI if the relation keyword is involved in either *dobj *or prep^* ^dependency. The "form" in the example in Figure 1-R4 is the only exception; it is allowed only if it has "complex" as its *dobj*. The negation is handled in the same way as Rule 1.

***Rule 5: ****P_i _- *{-, /} *- P_j _- *{***interaction, complex, heterodimer, product, assembly***}

This rule defines a lexical pattern that matches phrases such as "*P*0*-P*1 complex" and "*P*0*/P*1 heterodimer."

The negation is handled in the same way as Rule 2 and 3.

***Rule 6: ****P_i _- VERB - *{***receptor, ligand, substrate, binding protein***} ***- ***{***for, of***} *- P_j_*

This rule is constructed using both syntactic and lexical relations. A sentence like "*P*0 is a receptor for *P*1" implies that *P*0 will bind to *P*1. We identify four such keywords as above that imply the binding property between the two proteins. The negation is handled in the same way as Rule 1.

***Rule 7: ****P_i _- *{***receptor, ligand, substrate, binding protein***} *- *(*P_j_*)

In the example, "erythropoietin receptor (EPOR)," we know that EPOR is a protein that acts as a receptor for erythropoietin. We capture such relation using this simple lexical rule. The same relation holds for ligand and substrate. No negation handling is necessary in this rule.

***Rule 8: ****P_i _- *{***binding domain, binding site***} *- *{***in, within, on, of***} *- P_j_*

This is a sister rule to Rule 7, defining a similar relation using "binding domain" and "binding site." Like the rule above, it is a handy lexical pattern that matches high precision binding relations. For example, "CD30L binding domain on the human CD30 molecule" suggests a PPI between CD30L and CD30. No negation handling is required in this rule.

## Results

### Datasets

In order to test the performance of our approach, we use AIMed [9] corpus, which is considered as the *de facto *standard for the PPI extraction benchmark. There also exist several other benchmark corpora but we decided to use only AIMed for several reasons. Pyysalo et al [25] conducted a comparative analysis of five popular PPI corpora including AIMed [9], BioInfer [26], HPRD50 [2], IEPA [27], and LLL [28]. They reported that there are sizable discrepancies among the corpora, which are introduced mainly by the differences in annotation policy.

Unlike others, AIMed strictly focuses on causal relations that lead to physical changes or changes in dynamics in the target molecule. The other corpora include non-causal interactions such as *part-of *and *is-a *relations. For example, BioInfer annotates static relations like protein family memberships, as interactions. Some corpora are more inclusive while some others are more restrictive in interaction determination. Because of these differences, the number of annotated interactions and the level of certainty differ widely across the corpora. We chose AIMed because we focus only on the causal relations and aim to extract only highly certain relations; AIMed turns out the best-fit benchmark for our purpose.

### Baselines

We compare our approach with two state-of-the-art PPI extraction methods: a tree kernel-based method [20] and a hybrid method [6]. The tree kernel-based method uses a subset tree kernel for learning [17]. The hybrid method works in two steps. In the first phase, it groups candidate PPI pairs by applying five hand crafted template patterns and in the second phase, it trains an SVM per group using a different set of features in each group. The performances of the two methods are reported as comparable to other current state-of-the-art methods.

### Evaluation method

As mentioned earlier, we argue that we need a new way to measure the PPI extraction performance. In this work, we introduce a "per-relation" basis performance evaluation method. The key idea behind this new evaluation method has been discussed already in the introduction section. Now we formulate the per-relation evaluation metrics. Let $\hat{TP}$ denote the number of true positive relations, $\hat{FP}$, the false positives, and $\hat{FN}$, the false negatives. While the conventional "per-instance" *TP *and *FP *are computed by counting the number of correctly predicted relation instances, our "per-relation" $\hat{TP}$ and $\hat{FP}$ are computed by counting the number of distinct protein pairs that are predicted correctly.

The question here is by what criteria we decide the interaction and non-interaction for a pair. A viable solution is a sophisticated weighted vote based on the prediction labels assigned to each instance of the relation [29]. However, in this work, we use a simple strategy where one positive instance makes the corresponding relation positive. We leave the investigation for a more sophisticated voting scheme for future work because it is out of scope of the current work. Finally, we turn to define the per-relation precision, recall, and F-score as follows.

(1) $$Precision=\frac{\hat{TP}}{\hat{TP}+\hat{FP}}$$

(2) $$Recall=\frac{\hat{TP}}{\hat{TP}+\hat{FN}}$$

(3) $$F-score=\frac{2\times Recall\times Precision}{Recall+Precision}$$

### Performance of our rule-based algorithm as a stand-alone extraction system

Table 2 shows the experiment results. Our approach achieved the highest precision in both per-relation and per-instance evaluations. We conducted the test by varying the minimum number of instances per relation (hereafter, *MIpR*). For example, the first group (*MIpR *= 1) represents experiment where we test relations with at least *one *instance (i.e., all relations; equal to full AIMed corpus). Similarly, the second group (*MIpR *= 2) represents the experiments involving only the relations with at least *two *instances. In order to test the effects of the number of positive instances on the classification accuracy for a positive relation, we further constrained the number of positive instances per positive relation to be at least two in the second group (likewise, three in the third group, etc.). We need this additional constraint because even for a positive PPI relation, there are many negatively annotated instances for it. In AIMed, there are total of 618 positive and 2312 negative relations (not instances). A positive relation contains on average 1.6 positive and 1.5 negative instances.

**Table 2:** Per-relation and per-instance performance evaluation results

Min #Inst. per Relation	#Uniq. Relations		#Instances		Methods	Per-Relation					Per-Instance				
			
	Pos	Neg	Pos	Neg		TP	FP	Precision	Recall	F-Score	TP	FP	Precision	Recall	F-Score
1	618	2,312	1,000	4,834	ours	132	7	**0.95**	0.214	0.349	153	10	**0.939**	0.153	0.263
					hybrid [6]	477	344	0.581	**0.772**	**0.663**	694	584	0.543^1^	**0.694**^1^	**0.609**^1^
					SST-PT [20]	195	102	0.657	0.316	0.427	217	170	0.561^2^	0.217^2^	0.313^2^

2	197	695	579	2,827	ours	80	3	**0.964**	0.406	0.571	101	5	**0.953**	0.174	0.294
					hybrid [6]	183	164	0.527	**0.929**	**0.673**	389	327	0.543	**0.672**	**0.601**
					SST-PT [20]	103	71	0.592	0.523	0.555	131	113	0.537	0.226	0.318

3	89	314	363	1,835	ours	51	2	**0.962**	0.573	**0.718**	67	3	**0.957**	0.185	0.310
					hybrid [6]	88	98	0.473	**0.989**	0.640	250	236	0.514	**0.689**	**0.589**
					SST-PT [20]	56	47	0.544	0.629	0.583	85	79	0.518	0.234	0.322

4	51	181	249	1,349	ours	35	2	**0.946**	0.686	**0.795**	50	3	**0.943**	0.201	0.331
					hybrid [6]	49	67	0.422	**0.961**	0.587	160	170	0.485	**0.643**	**0.553**
					SST-PT [20]	35	30	0.538	0.686	0.603	62	54	0.534	0.249	0.340

5	28	107	157	984	ours	23	1	**0.958**	0.821	**0.884**	37	1	**0.974**	0.236	0.380
					hybrid [6]	28	44	0.389	**1**	0.560	105	105	0.500	**0.669**	**0.572**
					SST-PT [20]	21	15	0.583	0.75	0.656	37	30	0.552	0.236	0.331

**Figure 2:**
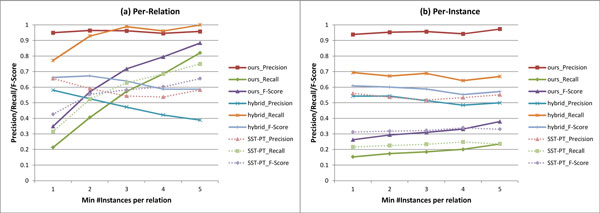
**Per-relation and per-instance evaluation results with varying "min #instance per relation"**.

As shown in Table 2, our rule-based approach exhibits the highest precision in all groups in both per-relation and per-instance evaluations. The per-relation precision is around 95-96% and the per-instance precision is around 94-97%. As expected (and intended so), the per-instance recall is not impressive achieving only 15-24%. On the other hand, the per-relation recall gradually improves up to 82% in group 5. We note that the baseline approaches tradeoff the per-relation precision for the per-relation recall as they move on from group 1 to group 5. Contrastingly, our approach does not degrade the precision while achieving substantial improvement on recall as it move on to group 5. Figure 2 illustrates the performance changes over the varying minimum instance requirements. These results suggest that our high-precision approach is more appropriate than the baseline approaches, especially for a use case involving large corpora.

### Improving the performance of baselines with our method

We wanted to test if our high-precision method can be augmented to an existing PPI extraction tool, in order to improve the performance of the original method. In Table 3, the first and the third rows represent the (per-instance) performances of the original baselines. The second row represents the result obtained from pipelining the hybrid baseline and our rule-based method. The pipelining is done as follows. We run the hybrid method to completion and save the instances that are predicted positive. We then run our method over those instances rejected by the hybrid method and finally compute the overall TP and FP by aggregating the numbers obtained from the first and the second runs. As we can see, the performance of the hybrid baseline, though marginal, was improved.

**Table 3:** Performance improvement of baselines through pipelining our approach

	TP	FP	**Prec**.	**Rec**.	F
**Hybrid**	694	584	0.543	0.694	0.609
**+ours** (%increase)	713 (2.7%)	585 (0.2%)	0.549 (1.2%)	0.713 (2.7%)	0.621 (1.8%)
**SST-PT**	217	170	0.561	0.217	0.313
**+ours** (%increase)	322 (48.4%)	178 (4.7%)	0.644 (14.8%)	0.322 (48.4%)	0.429 (37.1%)

The performance improvement was much more significant with SST-PT. The precision, recall, and F-score were improved by 15%, 48%, and 37%, respectively. The result suggests that our high-precision method can be also useful even in the conventional "per-instance" basis evaluation scenarios as we can achieve easy improvements of the performance of existing tools through the simple pipelining of our method.

### Performance of our rule-based method as a two-tier extraction system

Motivated by the positive results from the pipelining approach in the previous section, we generalize our extraction framework to a two-tier system. Our rule-based method can be placed either in the first tier or in the second tier while plugging in an existing PPI tool in the remaining tier. This two-tier system has many possible materializations; for example, in the first tier, we run our high-precision rule-based extraction method and pipeline all the negatively labeled instances to an SVM classifier in the second tier for an additional screening.

We evaluated several popular machine learning models for our two-tier system including Support Vector Machine (SVM), Naive Bayesian (NB), Decision Tree (DT), and k-Nearest Neighbor (kNN), in addition to the two baseline PPI extraction tools. For the machine learning models, as features we used two sets of bigrams taken from a forward dependency chain which is a dependency path from the root of a dependency tree to the first protein and from a backward chain which is a dependency path from the root to the second protein. For improving the accuracy and efficiency of the machine learning models, we performed feature selection where we picked the top-100 bigrams from each of the two bigram sets (i.e., sets of bigrams taken from the forward and the backward chains) based on the differences between their occurrences in the forward chains and the backward chains; for example, the top-100 forward bigrams are selected after sorting the bigrams based on the number of the occurrences in the forward chains subtracted by the number of the occurrences of the corresponding bigrams in the backward chains.

Table 4 shows the results of the experiments where our rule-based system is put in the first tier. For example, with the *MIpR *being set to one, the "ours+hybrid" model achieved the per-relation precision, recall, and F-score of 0.594, 0.772, and 0.671, respectively. It represents a slight improvement from the stand-alone hybrid model shown in Table 2 (2.2%, 0%, 1.2% from 0.581, 0.772, 0.663, respectively). Similarly, per-instance performance also was improved by 2.8%, 1.0%, 2.0% from 0.543, 0.694, 0.609 to 0.558, 0.701, 0.621, respectively. Similar improvements were observed in all five MIpR settings from 1 to 5 while the gain gradually increased as the MIpR increased. For example, with *MIpR *= 5, the per-relation performance was improved by 5.9%, 0%, 4.3% from 0.389, 1, 0.560 to 0.412, 1, 0.584, respectively. The per-instance performance was also improved by 10.2%, 2.8%, 7.0% from 0.5, 0.669, 0.572 to 0.551, 0.688, 0.612, respectively.

**Table 4:** Performance of the two-tier extraction system: our rule-based system in the first tier

Min #Inst. per Relation	#Uniq. Relation		#Instances		Methods	Per-Relation					Per-Instance				
			
	Pos	Neg	Pos	Neg		TP	FP	Precision	Recall	F-Score	TP	FP	Precision	Recall	F-Score
					ours	132	7	**0.950**	0.214	0.349	153	10	**0.939**	0.153	0.263
					+hybrid	477	326	0.594	**0.772**	**0.671**	701	555	0.558	**0.701**	**0.621**
					+SST-PT	231	112	0.673	0.374	0.481	287	167	0.632	0.287	0.395
1	618	2,312	1,000	4,834	+SVM	152	44	0.776	0.246	0.374	172	67	0.720	0.172	0.278
					+NB	247	254	0.493	0.400	0.442	285	410	0.410	0.285	0.336
					+DT	155	40	0.795	0.251	0.382	180	67	0.729	0.180	0.289
					+kNN	215	226	0.488	0.348	0.406	242	310	0.438	0.242	0.312

					ours	80	3	**0.964**	0.406	0.571	101	5	**0.953**	0.174	0.294
					+hybrid	183	151	0.548	**0.929**	**0.689**	395	310	0.560	**0.682**	**0.615**
					+SST-PT	127	62	0.672	0.645	0.658	186	102	0.646	0.321	0.429
2	197	695	579	2,827	+SVM	94	36	0.723	0.477	0.575	116	63	0.648	0.200	0.306
					+NB	113	110	0.507	0.574	0.538	151	196	0.435	0.261	0.326
					+DT	90	21	0.811	0.457	0.585	112	45	0.713	0.193	0.304
					+kNN	129	138	0.483	0.655	0.556	163	235	0.410	0.282	0.334

					ours	51	2	**0.962**	0.573	0.718	67	3	**0.957**	0.185	0.310
					+hybrid	88	94	0.484	**0.989**	0.650	257	222	0.537	**0.708**	**0.611**
					+SST-PT	70	38	0.648	0.787	0.711	129	58	0.690	0.355	0.469
3	89	314	363	1,835	+SVM	58	22	0.725	0.652	0.687	78	32	0.709	0.215	0.330
					+NB	59	54	0.522	0.663	0.584	86	89	0.491	0.237	0.320
					+DT	53	5	0.914	0.596	**0.722**	70	8	0.897	0.193	0.318
					+kNN	66	85	0.437	0.742	0.550	97	153	0.388	0.267	0.316

					ours	35	2	**0.946**	0.686	**0.795**	50	3	**0.943**	0.201	0.331
					+hybrid	51	63	0.447	**1**	0.618	171	150	0.533	**0.687**	**0.600**
					+SST-PT	45	27	0.625	0.882	0.732	92	43	0.681	0.369	0.479
4	51	181	249	1,349	+SVM	38	15	0.717	0.745	0.731	55	25	0.688	0.221	0.335
					+NB	42	43	0.494	0.824	0.618	67	86	0.438	0.269	0.333
					+DT	36	5	0.878	0.706	0.783	54	8	0.871	0.217	0.347
					+kNN	44	56	0.440	0.863	0.583	74	104	0.416	0.297	0.347

					ours	23	1	**0.958**	0.821	**0.884**	37	1	**0.974**	0.236	0.380
					+hybrid	28	40	0.412	**1**	0.584	108	88	0.551	**0.688**	**0.612**
					+SST-PT	25	14	0.641	0.893	0.746	57	22	0.722	0.363	0.483
5	28	107	157	984	+SVM	25	12	0.676	0.893	0.769	40	18	0.690	0.255	0.372
					+NB	24	21	0.533	0.857	0.657	40	40	0.500	0.255	0.338
					+DT	23	4	0.852	0.821	0.836	37	8	0.822	0.236	0.367
					+kNN	26	31	0.456	0.929	0.612	45	51	0.469	0.287	0.356

The improvement of the stand-alone SST-PT model was even more drastic. With *MIpR *= 5, SST-PT's per-relation performance was improved by 9.9%, 19.1%, 13.7% from 0.583, 0.75, 0.656 to 0.641, 0.893, 0.746, respectively, while its per-instance performance was improved by striking percentages of 30.8%, 53.8%, 45.9% from 0.552, 0.236, 0.331 to 0.722, 0.363, 0.483, respectively.

On the other hand, the two-tier approach turned out to be not as effective for improving the performance against our stand-alone rule-based system as it was for improving the baselines. For example, with *MIpR *= 5, the per-relation performance of our method after pipelining to the hybrid model was improved by -133%, 21.8%, -51.4% from 0.958, 0.821, 0.884 to 0.412, 1, 0.584, for precision, recall, F-score, respectively. Similarly, the per-instance performance was improved by -76.8%, 192%, 61.1% from 0.974, 0.236, 0.38 to 0.551, 0.688, 0.612, respectively. The two-tier approach generally improved the per-relation and per-instance recall of our rule-based model while substantially degrading its precision. Similar observations were made across the models.

In this test, we only used one type of feature, the dependency bigram, for the machine learning models. In order to see if we can improve the performance further by adding more features to the models, we conducted an additional test as shown in Table 5. We only show the result of the "ours+SVM" model in this test. The remaining models showed the similar performance characteristic as that of the "ours+SVM" model. The first row in each *MIpR *group represents the result of "ours+SVM" with the top-100 forward and backward dependency bigrams. The second row represents the result produced with an additional feature of dependency length which is the length of a dependency path from the root of a dependency tree to a protein. We used both forward and backward dependency lengths. The third row shows the result with an offset distance between two proteins in a sentence added in addition to the original dependency bigrams. The fourth row shows the result from using all three types of features including the dependency bigrams, the dependency lengths, and the offset distance. As we can see in the table, however, the improvement that we achieved by adding more features was not significant. Only a slight improvement on precision was achieved by using the dependency lengths together with the dependency bigrams.

**Table 5:** Performance of the "ours+SVM" model with incremental feature sets

Min #Inst. per Relation	#Uniq. Relation		#Instances		Methods	Per-Relation					Per-Instance				
			
	Pos	Neg	Pos	Neg		TP	FP	Precision	Recall	F-Score	TP	FP	Precision	Recall	F-Score
1	618	2,312	1,000	4,834	ours+SVM	152	44	0.776	**0.246**	**0.374**	172	67	0.720	0.172	0.278
					+dep. len.	149	35	**0.810**	0.241	0.371	170	52	**0.766**	0.170	0.278
					+dist.	150	39	0.794	0.243	0.372	171	63	0.731	0.171	0.277
					+both	151	38	0.799	0.244	**0.374**	173	58	0.749	**0.173**	**0.281**

2	197	695	579	2,827	ours+SVM	94	36	0.723	**0.477**	0.575	116	63	0.648	0.200	0.306
					+dep. len.	94	32	**0.746**	**0.477**	**0.582**	117	53	0.688	**0.202**	**0.312**
					+dist.	92	35	0.724	0.467	0.568	117	54	0.684	**0.202**	**0.312**
					+both	94	32	**0.746**	**0.477**	**0.582**	116	51	**0.695**	0.200	0.311

3	89	314	363	1,835	ours+SVM	58	22	**0.725**	**0.652**	**0.687**	78	32	0.709	**0.215**	**0.330**
					+dep. len.	57	22	0.722	0.640	0.679	76	31	**0.710**	0.209	0.323
					+dist.	57	22	0.722	0.640	0.679	75	31	0.708	0.207	0.320
					+both	57	22	0.722	0.640	0.679	76	31	**0.710**	0.209	0.323

4	51	181	249	1,349	ours+SVM	38	15	0.717	**0.745**	**0.731**	55	25	0.688	**0.221**	**0.335**
					+dep. len.	36	13	**0.735**	0.706	0.720	52	19	**0.732**	0.209	0.325
					+dist.	38	15	0.717	**0.745**	**0.731**	55	25	0.688	**0.221**	**0.335**
					+both	36	13	**0.735**	0.706	0.720	52	19	**0.732**	0.209	0.325

5	28	107	157	984	ours+SVM	25	12	0.676	**0.893**	0.769	40	18	0.690	**0.255**	0.372
					+dep. len.	25	11	**0.694**	**0.893**	**0.781**	40	15	**0.727**	**0.255**	**0.378**
					+dist.	25	12	0.676	**0.893**	0.769	40	18	0.690	**0.255**	0.372
					+both	25	11	**0.694**	**0.893**	**0.781**	40	15	**0.727**	**0.255**	**0.378**

^1^Note that the result shown here is different from the ones reported in [6]. It may be due to the differences in SVM optimization parameters used for the experiments. We obtained the codes from the authors' web page at http://staff.science.uva.nl/$\tilde{\text{b}}$ui/PPIs.zip and ran as is with the parameters: RBF kernel gamma - 0.0145; C = 9; Weka Cost-SensitiveClassifier optimization.

^2^In [20], the authors reported the macro-averaged precision, recall, and F-score, which are incomparable to other performance results. Following the general convention in PPI research, we compared the performance using the precision, recall and F-score computed with only positive class prediction results. The original implementation was not available. We implemented it on SVM-LIGHT-TK ver 1.2 obtained from http://disi.unitn.it/moschitti/Tree-Kernel.htm. The optimization parameters used are C = 8 and λ = 0.6 (as reported in [20])

Finally, we conducted the same experiment as Table 4 after swapping the tiers. In this test, the two baseline models and the four machine learning models are used as the first tier system and our rule-based model is used as the second tier system. The result is shown in Table 6. The performance of the baselines was improved in both per-relation and per-instance evaluations as we pipelined the negatively-labeled instances to our rule-based system. For example, with *MIpR *= 5, the per-relation performance of the SST-PT method was improved by 4.6%, 19.1%, 10.5% from 0.583, 0.75, 0.656 to 0.61, 0.893, 0.725, respectively. The per-instance performance was also improved by 23.2%, 78.0%, 56.8% from 0.552, 0.236, 0.331 to 0.68, 0.42, 0.519, respectively. However, the two-tier approach was not as effective for improving our rule-based system as it was for the baselines. Similar to the previous experiment in Table 4, the two-tier system degrades the precision while improving the recall.

**Table 6:** Performance of the two-tier extraction system: our rule-based system in the second tier

Min #Inst. per Relation	#Uniq. Relation		#Instances		Methods	Per-Relation					Per-Instance				
			
	Pos	Neg		Neg		TP	FP	Precision	Recall	F-Score	TP	FP	Precision	Recall	F-Score
1	618	2,312	1,000	4,834	ours	132	7	**0.950**	0.214	0.349	153	10	**0.939**	0.153	0.263
					hybrid+	479	344	0.582	**0.775**	**0.665**	705	584	0.547	**0.705**	**0.616**
					SST-PT+	257	108	0.704	0.416	0.523	322	178	0.644	0.322	0.429
					SVM+	131	57	0.697	0.212	0.325	140	84	0.625	0.140	0.229
					NB+	235	247	0.488	0.380	0.427	261	410	0.389	0.261	0.312
					DT+	157	73	0.683	0.254	0.370	169	120	0.585	0.169	0.262
					kNN+	219	260	0.457	0.354	0.399	237	366	0.393	0.237	0.296

2	197	695	579	2,827	ours	80	3	**0.964**	0.406	0.571	101	5	**0.953**	0.174	0.294
					hybrid+	183	164	0.527	**0.929**	**0.673**	399	327	0.550	**0.689**	**0.612**
					SST-PT+	132	73	0.644	0.670	0.657	203	118	0.632	0.351	0.451
					SVM+	88	48	0.647	0.447	0.529	106	78	0.576	0.183	0.278
					NB+	107	113	0.486	0.543	0.513	140	230	0.378	0.242	0.295
					DT+	88	41	0.682	0.447	0.540	104	85	0.550	0.180	0.271
					kNN+	125	151	0.453	0.635	0.529	152	250	0.378	0.263	0.310

3	89	314	363	1,835	ours	51	2	**0.962**	0.573	**0.718**	67	3	**0.957**	0.185	0.310
					hybrid+	88	98	0.473	**0.989**	0.640	262	236	0.526	**0.722**	**0.609**
					SST-PT+	71	48	0.597	0.798	0.683	136	82	0.624	0.375	0.468
					SVM+	43	28	0.606	0.483	0.538	59	40	0.596	0.163	0.256
					NB+	59	69	0.461	0.663	0.544	82	113	0.421	0.226	0.294
					DT+	49	17	0.742	0.551	0.632	61	26	0.701	0.168	0.271
					kNN+	59	86	0.407	0.663	0.504	84	151	0.357	0.231	0.281

4	51	181	249	1,349	ours	35	2	**0.946**	0.686	**0.795**	50	3	**0.943**	0.201	0.331
					hybrid+	50	67	0.427	**0.980**	0.595	171	171	0.500	**0.687**	**0.579**
					SST-PT+	45	32	0.584	0.882	0.703	98	57	0.632	0.394	0.485
					SVM+	33	19	0.635	0.647	0.641	47	35	0.573	0.189	0.284
					NB+	41	53	0.436	0.804	0.565	64	116	0.356	0.257	0.299
					DT+	32	7	0.821	0.627	0.711	43	11	0.796	0.173	0.284
					kNN+	39	61	0.390	0.765	0.517	59	118	0.333	0.237	0.277

5	28	107	157	984	ours	23	1	**0.958**	0.821	**0.884**	37	1	**0.974**	0.236	0.380
					hybrid+	28	44	0.389	**1**	0.560	114	105	0.521	**0.726**	**0.607**
					SST-PT+	25	16	0.610	0.893	0.725	66	31	0.680	0.420	0.519
					SVM+	21	14	0.600	0.750	0.667	29	22	0.569	0.185	0.279
					NB+	23	41	0.359	0.821	0.500	35	73	0.324	0.223	0.264
					DT+	18	3	0.857	0.643	0.735	26	4	0.867	0.166	0.279
					kNN+	24	38	0.387	0.857	0.533	35	68	0.340	0.223	0.269

## Discussion

As we can see from the results, our high-precision rule-based system is quite effective in many aspects especially for use cases involving large corpora. The results show that our rule-based system not only performs well as a stand-alone system but also serves well as a complement to other existing PPI extraction models. The latter property is important as our rule-based system can improve the performance of existing solutions simply by pipelining the existing solutions to ours without having to modify the internals of the other existing tools.

We also demonstrated that the generalized two-tier platform for PPI extraction is a viable alternative. The two-tier system can be useful for improving the performance of legacy PPI tools and also can be useful for use cases where recall is equally important. The remaining challenge is how we can retain the high precision of our rule-based system while improving its recall in the two-tier system. This seems to be an inherently difficult problem. The extraction model in the other tier should be extremely conservative in determining positive instances in order to retain the high precision. The six models we tested in this experiments all failed to achieve this goal. We leave this as our future work.

## Conclusion

In this work, we argued that the current "per-instance" basis performance evaluation method is not pragmatic in many realistic PPI extraction scenarios. To address this problem, we introduced a new "per-relation" basis evaluation method. In the new method, precision and recall are computed based on the number of distinct relations (not instances) that are classified correctly. We also proposed a high-precision rule-based PPI extraction method and showed our method achieves substantially higher precision than two state-of-the-art PPI extraction baselines in both per-relation and per-instance evaluation. Finally, we generalized our rule-based model to a two-tier PPI extraction system, in which our rule-based model is augmented with other existing extraction models through pipelining. With this two-tier system, we demonstrated that our rule-based model is also a valuable complement to other existing PPI tools. In our future work, we plan to investigate more sophisticated weighted voting scheme in order to make our PPI extraction system more robust to potential parsing and annotation errors. We also plan to investigate highly conservative high-precision machine learning models in order to retain the high precision of our rule-based system while improving the recall when used in our two-tier system.

## Authors' contributions

JK carried out the design of the system and drafted the manuscript. JL and SK participated in the implementation of the system and its validation. SL and KL carried out the use of the system for validation and helped to draft the manuscript. All authors read and approved the final manuscript.

## Competing interests

The authors declare that there are no competing interests.

## Declarations

The publication costs for this article were funded by the Next-Generation Information Computing Development Program through the National Research Foundation of Korea (NRF) funded by the Ministry of Education, Science and Technology (2012M3C4A7033341).

This article has been published as part of *BMC Medical Informatics and Decision Making *Volume 13 Supplement 1, 2013: Proceedings of the ACM Sixth International Workshop on Data and Text Mining in Biomedical Informatics (DTMBio 2012). The full contents of the supplement are available online at http://www.biomedcentral.com/bmcmedinformdecismak/supplements/13/S1.
